# Reduced levels of SCD1 accentuate palmitate-induced stress in insulin-producing β-cells

**DOI:** 10.1186/1476-511X-9-108

**Published:** 2010-09-29

**Authors:** Kristofer Thörn, Meri Hovsepyan, Peter Bergsten

**Affiliations:** 1 Department of Medical Cell Biology, Uppsala University, BMC Box 571, 75123 Uppsala, Sweden; 2 Institute of Molecular Biology of Armenian National Academy of Sciences, 7 Hasratyan St, 0014 Yerevan, Republic of Armenia

## Abstract

**Background:**

Stearoyl-CoA desaturase 1 (SCD1) is an ER resident enzyme introducing a double-bond in saturated fatty acids. Global knockout of SCD1 in mouse increases fatty acid oxidation and insulin sensitivity which makes the animal resistant to diet-induced obesity. Inhibition of SCD1 has therefore been proposed as a potential therapy of the metabolic syndrome. Much of the work has focused on insulin target tissue and very little is known about how reduced levels of SCD1 would affect the insulin-producing β-cell, however. The aim of the present study was therefore to investigate how reduced levels of SCD1 affect the β-cell.

**Results:**

Insulin-secreting MIN6 cells with reduced levels of SCD1 were established by siRNA mediated knockdown. When fatty acid oxidation was measured, no difference between cells with reduced levels of SCD1 and mock-transfected cells were found. Also, reducing levels of SCD1 did not affect insulin secretion in response to glucose. To investigate how SCD1 knockdown affected cellular mechanisms, differentially regulated proteins were identified by a proteomic approach. Cells with reduced levels of SCD1 had higher levels of ER chaperones and components of the proteasome. The higher amounts did not protect the β-cell from palmitate-induced ER stress and apoptosis. Instead, rise in levels of p-eIF2α and CHOP after palmitate exposure was 2-fold higher in cells with reduced levels of SCD1 compared to mock-transfected cells. Accordingly, apoptosis rose to higher levels after exposure to palmitate in cells with reduced levels of SCD1 compared to mock-transfected cells.

**Conclusions:**

In conclusion, reduced levels of SCD1 augment palmitate-induced ER stress and apoptosis in the β-cell, which is an important caveat when considering targeting this enzyme as a treatment of the metabolic syndrome.

## Background

Stearoyl-CoA desaturases (SCD:s) are a family of endoplasmic reticulum (ER) resident enzymes introducing a Δ9 double-bond in saturated fatty acids, thereby generating their monounsaturated counterparts [1]. In mice, four isoforms of SCD have been characterized, which all catalyze the same reaction but have somewhat different substrate specificities [1]. The physiological role of having multiple isoforms is not fully understood, but the different expression patterns and inducibility indicate exclusive roles for the different isoforms [2]. In humans, only one functional ortholog to mouse SCD has been found [3]. In addition, humans express another SCD isoform, termed hSCD5, which is unique to primates [4]. Much focus has been directed towards the SCD1 isoform, which is ubiquitously expressed in mouse, and the major isoform found in liver and adipose tissue [2]. While SCD1 seems to have a protective effect in many cell types exposed to saturated fatty acids *in vitro *[5-7], evidence from SCD1 KO mice and from mice injected with antisense oligonucleotides against SCD1 has shown that lack of SCD1 protects the animals from diet-induced obesity [8,9]. The lack of SCD1 results in higher energy expenditure, reduced fatty acid *de novo *synthesis, decreased expression of lipogenic genes, and increased insulin sensitivity [8,10]. In human studies, elevated levels of SCD1 were positively correlated with high triglyceride levels in familial hypertriglyceridemia subjects [11], increased body mass index, reduced fatty acid oxidation and high plasma insulin levels [12]. It has been suggested that SCD1 acts as a main molecular switch between lipolysis and lipogenesis, as increased SCD1 expression precedes the increased expression of other lipogenic genes in mice fed a diet high in stearate. The increased expression of SCD1 also coincides with an increase in SREBP-1c and PPAR-γ coactivator-1β (PGC-1β) [13]. Reducing or inhibiting the enzyme has therefore been proposed as a novel treatment for obesity, type-2 diabetes mellitus and related metabolic disorders [14]. Accordingly, efforts have been made to identify pharmacological inhibitors of SCD1. In agreement with results obtained in the SCD1 KO mouse [9], oral administration of a selective SCD1 inhibitor strongly repressed the diet-induced weight gain in C57BL6 mice as well as decreased the desaturation index (oleate/stearate) [15]. Also, the effects of reduced SCD1 were reproduced in rat models where SCD1 inhibition reduced plasma triglyceride levels and improved insulin sensitivity [16]. Work regarding the role of SCD1 has primarily focused on insulin target tissue such as liver, muscle and white adipose tissue. Very little is known about the role of the enzyme in the insulin-producing pancreatic β-cell and how reduced SCD1 levels would affect the cell. The aim of the present study was to test if reducing the levels of SCD1 had positive effects also on the β-cell, and if so, by which pathways these effects were mediated.

## Results

### Fatty acid oxidation and insulin secretion in MIN6 cells with reduced levels of SCD1

**Figure 1:**
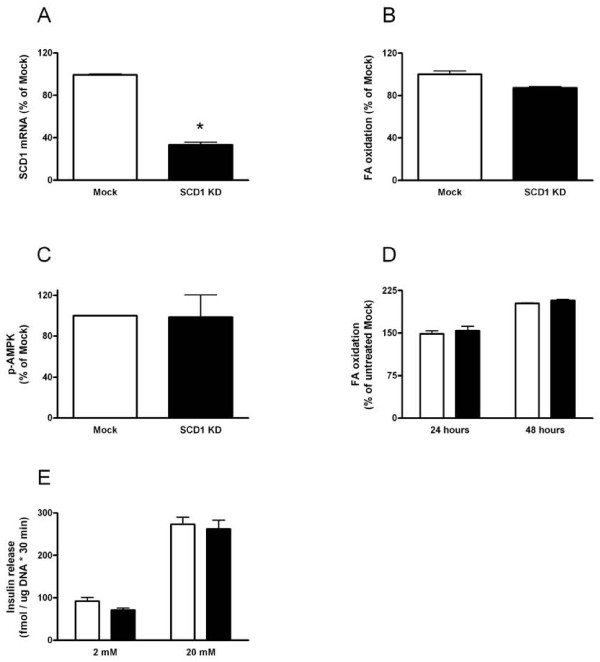
**SCD1 mRNA levels, fatty acid oxidation and glucose-stimulated insulin secretion in MIN6 cells transfected with SCD1 siRNA**. MIN6 cells were transfected with siRNA targeted towards SCD1 (black bar) or with nonsense siRNA (Mock; white bar). After 72 hours culture, SCD1 transcript levels were measured (panel A). Fatty acid oxidation (panel B) and levels of p-AMPK (panel C) were measured 72 hours post-transfection in mock-transfected and SCD1 KD cells. Fatty acid oxidation was measured after 24 or 48 hours exposure to 0.25 mM palmitate (panel D). MIN6 cells transfected with mock siRNA or SCD1 siRNA were stimulated with 2 or 20 mM glucose. After stimulation, secreted insulin was measured (panel E). Results are means ± SEM from 4-6 individual experiments. *P < 0.05 denotes change compared to mock-transfected cells.

Insulin-producing MIN6 cells with reduced SCD1 levels were obtained by knocking down (KD) the levels of the enzyme with siRNA (SCD1 KD cells). Transfection with SCD1 siRNA efficiently reduced transcript levels, which were 37% of levels in cells transfected with nonsense siRNA (mock-transfected cells) 72 hours post transfection (Fig 1A).

A hallmark of the SCD1 KO mouse is increased fatty acid oxidation and phosphorylation of AMPK, which are suggested to contribute to explain the resistance to diet-induced weight gain in these animals [17]. To test if these results were reproducible also in MIN6 cells with lowered SCD1, fatty acid oxidation and phosphorylation of AMPK were measured in SCD1 KD and mock-transfected cells. No difference in level of fatty acid oxidation between cells with reduced levels of SCD1 and control cells could be detected, however (Fig 1B). Also, when phosphorylated AMPK was measured, similar levels were obtained in mock-transfected and SCD1 KD cells (Fig 1C). The SCD1 KO mouse has increased fatty acid oxidation also when fed a diet rich in saturated fatty acids [13]. When we measured fatty acid oxidation in SCD1 KD and mock-transfected cells exposed to palmitate for 24 or 48 hours, no difference between SCD1 KD and mock-transfected cells in levels of fatty acid oxidation could be detected (Fig 1D).

The primary function of the pancreatic β-cell is to secrete insulin in response to elevated nutrient levels, primarily glucose. The SCD1 KO mouse has increased insulin receptor signaling [10]. Insulin receptors are present also on the β-cell and it has been shown that increased signaling potentiates insulin secretion [18]. We therefore tested if the secretory response to glucose was altered by reducing the levels of SCD1. Glucose-stimulated insulin secretion rose 3-fold in mock-transfected cells when increasing the glucose concentration from 2 to 20 mM (Fig 1E). Similar changes were observed in SCD1 KD cells, however. From the results we concluded that energy metabolism and function were not altered in MIN6 SCD1 KD cells in contrast to liver cells from the SCD1 KO mouse [17].

### Proteomic analysis on cellular proteins differently regulated by SCD1 KD

We next used a proteomic approach to identify mechanisms by which reduced SCD1 levels affected MIN6 cells. Cellular proteins obtained from SCD1 KD and mock-transfected cells were separated, quantified and differently expressed proteins identified. Knockdown of SCD1 mainly up-regulated proteins involved in protein folding and degradation (Table 1). Protein disulfide isomerase (PDI), prolyl 4-hydroxylase and glucose-regulated protein 94 (GRP94), chaperones involved in protein folding in the ER, were all up-regulated by more than 50% when SCD1 levels were lowered. Indication of increased expression of the proteasome was also found as two of its components, proteasome subunit α type 3 and proteasome 26 S subunit, were up-regulated by more than 50% in SCD1 KD cells compared to mock-transfected cells. In addition, levels of proteins involved in RNA modulation and serine metabolism were elevated, although the importance of these proteins was not evaluated.

**Table 1:** Cellular proteins differentially regulated by SCD1 knockdown.

Protein	SCD1 KD
Rho GDP dissociation inhibitor (GDI) α	36% up
Prolyl 4-hydroxylase, β	80% up
GRP94	64% up
Proteasome subunit α type 3	52% up
Proteasome 26 S subunit	75% up
Heterogeneous nuclear ribonucleoprotein H1	46% up
Protein disulfide isomerase associated 3	78% up
Nucleosome assembly protein	136% up
Seryl-aminoacyl-tRNA synthetase	93% up
Nucleolysin TIAR	144% up
Phosphoserine aminotransferase 1	73% up

MIN6 cells were transfected with siRNA targeted towards SCD1 or with nonsense siRNA (mock). After 72 hours culture, cellular proteins were isolated and subjected to 2D-gel electrophoresis. After staining, differentially expressed proteins were cut and identified using peptide mass fingerprint. Differences indicate change induced by reduction in SCD1 levels. Results are means of 4 independent experiments. Significantly different (P < 0.05) expressed proteins in SCD1 KD cells compared with mock transfected cells are shown.

### ER stress response and apoptosis in MIN6 cells with reduced levels of SCD1

**Figure 2:**
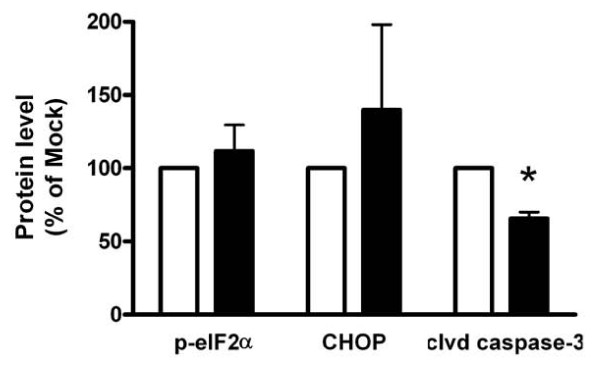
**Levels of p-eIF2α, CHOP and cleaved caspase-3 in MIN6 cells with reduced SCD1 levels**. MIN6 cells were transfected with siRNA targeted towards SCD1 (black bar) or with nonsense siRNA (mock; white bar). After 72 hours culture, protein levels of p-eIF2α, CHOP and cleaved caspase-3 were measured. Results are means ± SEM from 4-6 individual experiments. *P < 0.05 denotes difference compared to mock-transfected cells.

Enhanced chaperone and proteasome expression is part of the unfolded protein response (UPR), which is elicited when the ER load is enhanced and un- or misfolded proteins accumulate. When the UPR is not successful in alleviating the load on the ER, apoptosis is induced [19]. In β-cells, ER stress mainly connects to apoptosis through the PERK pathway via its downstream effectors phosphorylated eIF2α (p-eIF2α) and CHOP. As ER chaperones and proteasomal components were up-regulated after SCD1 knockdown, we hypothesized that PERK signaling would be decreased. When levels of p-eIF2α and expression of CHOP were measured there was no difference between SCD1 KD and mock-transfected cells cultured under control conditions (Fig 2). Knockdown of SCD1 caused a reduction in the cellular levels of cleaved caspase-3, however.

**Figure 3:**
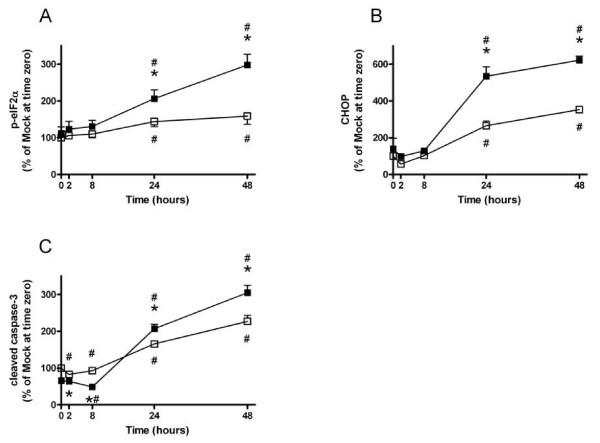
**Levels of markers of ER stress and apoptosis in SCD1 KD and mock-transfected MIN6 cells after palmitate exposure**. MIN6 cells were transfected with siRNA targeted towards SCD1 (black square) or with nonsense siRNA (mock; white square) and cultured for the indicated time periods in the presence of 0.25 mM palmitate. After culture, protein levels of p-eIF2α (panel A), CHOP (panel B), and cleaved caspase-3 (panel C) were measured. Results are means ± SEM from 3-6 individual experiments. *P < 0.05 denotes effect of SCD1 KD compared to mock-transfected cells at indicated time point and #P < 0.05 denotes effect of palmitate compared to time 0.

The lack of difference between SCD1 KD and mock-transfected cells on UPR could be accounted for by low levels of ER stress under control culture conditions. We therefore exposed MIN6 cells to the fatty acid palmitate, which elicits ER stress and induces apoptosis [20]. Indeed, levels of p-eIF2α rose after 24 hours of palmitate exposure (Fig 3A). Levels of p-eIF2α were higher in SCD1 KD cells than in mock-transfected cells after 24 and 48 hours of palmitate exposure, however. Phosphorylation of eIF2α precedes the alternative translation of ATF4, which in turn increases the amount of CHOP. In agreement with this temporal relationship, levels of CHOP began to rise after 24 hours of palmitate exposure in both mock-transfected and SCD1 KD cells (Fig 3B). Whereas CHOP levels in mock-transfected cells increased 3-fold after 48 hours of palmitate exposure, levels in SCD1 KD cells increased more than 6-fold. CHOP acts as a pro-apoptotic mediator in the cell, and increased levels of the protein have been associated with increased rates of apoptosis [21]. To examine if the increased levels of CHOP translated into apoptosis, we measured levels of cleaved caspase-3. Cells with reduced levels of SCD1 had lower levels of cleaved caspase-3 than mock-transfected cells during the first 8 hours of exposure to palmitate (Fig 3C). After exposure to the fatty acid for 24 hours, levels of cleaved caspase-3 increased in both SCD1 KD cells and mock-transfected cells. In agreement with elevated levels of CHOP, levels of cleaved caspase-3 were higher in SCD1 KD cells than in mock-transfected cells after 24 hours of palmitate exposure. In conclusion, reducing the levels of SCD1 in insulin-secreting MIN6 cells increased ER stress and apoptotic signaling in response to palmitate.

## Discussion

Reduction of SCD1 has beneficial effects in whole animal studies. The positive role of reducing [6] or completely removing SCD1 [7] has been linked to increased fatty acid oxidation and insulin sensitivity, and to a reduction of fatty acid *de novo *synthesis. Consequently, inhibiting SCD1 has been suggested as a potential therapy for the metabolic syndrome [16]. Administration of the SCD1 inhibitor GSK993 to a diet-induced insulin resistant rat model readily improved insulin sensitivity [16], and a beneficial phenotype has also been seen *in vivo *by infusing the animals with antisense oligonucleotides towards SCD1 [8]. In our *in vitro *model, the reduction of SCD1 did neither lead to an increase in fatty acid oxidation under control conditions, nor after exposure to palmitate for 24 or 48 hours. Also, no differences in insulin secretion were observed between cells with reduced levels of SCD1 and mock-transfected cells. The difference between results obtained from the global SCD1 KO mouse and the present results may be explained by experiences from the SCD1 skin-specific knockout (SKO) mouse [22], which is also resistant to diet-induced obesity. The SKO mouse has increased thermogenesis due to a damaged skin barrier and has reduced expression of lipogenic genes in the liver suggesting that the beneficial phenotype in the global knockout may at least partly depend on enhanced heat loss in these animals. The cellular response to ablation of SCD1 also seems to be tissue dependent. Cardiomyocytes from the SCD1 KO mouse have inhibited fatty acid oxidation and increased glucose utilization [23], while primary mouse hepatocytes transfected with antisense oligonucleotides towards SCD1 show decreased fatty acid synthesis and increased oxidation [8]. Also, white adipose tissue from the SCD1 KO mouse displays lower levels of inflammation and improved insulin signaling [24]. Since both hepatocytes and adipocytes are major players in lipogenesis, it could be postulated that the positive effects of SCD1 reduction or removal is primarily linked to lipogenic tissues.

To explore what effects reduced levels of SCD1 had in insulin-producing β-cells, we protein profiled cells with reduced and control SCD1 levels. In the SCD1 KD cells, levels of chaperones and proteasomal components were up-regulated, indicating increased capacity of the ER to handle client proteins. When cells were challenged with the fatty acid palmitate, rather than being protected from ER stress, they exhibited increased levels of p-eIF2α, CHOP and cleaved caspase-3, however. SCD1 is the enzyme responsible for conversion of the saturated fatty acid palmitate to its monounsaturated counterpart palmitoleate. Since exposure to palmitoleate does not elicit ER stress [25], the disruption in ER homeostasis may be accentuated in SCD1 KD cells exposed to palmitate. In agreement with this, it was shown that human myotubes have an inverse correlation between the susceptibility to develop palmitate-induced ER stress and the levels of SCD1 [7]. The β-cell has been shown to be particularly sensitive to ER stress, probably due to its high synthesis of insulin [26]. The phenomenon of enhanced sensitivity to ER stress in SCD1 KD cells may also be present in other cell types, however, as knocking down levels of SCD1 in HeLa cells causes increased ER stress and induction of apoptosis [27]. It was postulated that the negative effects of SCD1 inhibition is mediated via the pro-inflammatory action of saturated fatty acid species such as palmitate, which accumulate after SCD1 inhibition [28]. In agreement with this hypothesis, inclusion of anti-inflammatory ω3 poly-unsaturated fatty acids (PUFA) prevented not only deleterious effects of SCD1 inhibition [28], but also palmitate-induced apoptosis [29]. Thus, supplementation of ω3-PUFA in conjunction with inhibition of SCD1 may provide a useful strategy to avoid the negative effects of SCD1 inhibition.

## Conclusions

In summary, our data suggest that reducing the levels of SCD1 in the β-cell augments the effect of palmitate on ER stress and apoptosis. The findings may have implications to situations with increased circulating lipids and should be taken into consideration when targeting SCD1 as a treatment of the metabolic syndrome.

## Methods

### Cell culture

Mouse MIN6 cells, a kind gift from Professor Jun-Ichi Miyazaki, Osaka University, were maintained in DMEM supplemented with 15% FBS, 100 units/ml of penicillin, 100 μg/ml streptomycin (all from Invitrogen, Carlsbad, CA) and 50 μM β-mercaptoethanol at 37°C and 5% CO_2. _All experiments with MIN6 cells were performed between passages 21 and 28. During fatty acid (FA) exposure 0.5% FA-free BSA (Boehringer Mannheim GmbH, Mannheim, Germany) was added.

### Knockdown of SCD1 mRNA

MIN6 cells were reverse transfected with pooled siRNA from Invitrogen and Ambion (Ambion, Austin, TX), each at 50 nM or with mock siRNA from Ambion at 100 nM in an antibiotic free media using Lipofectamine 2000 (Invitrogen) according to manufacturer's instructions. After 24 hours, the media was changed and the cells were treated as indicated.

### Fatty acid preparation and cell treatment

Stock solutions containing palmitate (Sigma P-9767, St. Louis, MO) were prepared by dissolving the fatty acid in 50% ethanol to a final concentration of 100 mM. The stock solution was then diluted in culture medium with 0.5% FA-free BSA (Boehringer Mannheim GmbH) to a final concentration of 0.25 mM. Cells were cultured to 60-70% confluence and exposed to the fatty acid for up to 48 hours.

### Analysis of mRNA expression by real-time PCR

Total RNA were isolated from MIN6 cells using the ChargeSwitch Total Cell RNA kit (Invitrogen) according to manufacturer's instructions and reverse transcribed with SuperScript™ III First-Strand Synthesis System for RT-PCR (Invitrogen) using Oligo-dT primers. The real-time PCR was performed in 10 μl volume containing ~20 ng RNA equivalent, 0.5 μM forward and reverse primers and 5 μl Dynamo Capillary SYBR green qPCR kit (Finnzymes, Espoo, Finland). Primers used for the amplification are shown in Table 2. PCR products were quantified fluorometrically using SYBR Green, and normalized to the housekeeping gene β-actin and relative to the control according to the following formula: target amount = 2^-ΔΔ*Ct*^, where ΔΔ*Ct *= {[*Ct *(target gene) - *Ct *(*β*-actin)] - [*Ct *(control) - *Ct *(*β*-actin control)}.

**Table 2:** Primers used for real-time PCR.

Target	Forward primer	Reverse primer
β-actin	GTTACAGGAAGTCCCTCACC	GGAGACCAAAGCCTTCATAC
SCD1	CTTCTTGCGATACACTCTGG	TGAATGTTCTTGTCGTAGGG

### Separation and identification of proteins

Proteins obtained from cells with reduced or control SCD1 levels were solubilized, separated, quantified and identified essentially as described recently [30]. In short, MIN6 cells were lysed in buffer containing 1% Triton X-100, 1% SDS and protease inhibitor cocktail, homogenized by sonication and re-suspended in rehydration solution for the iso-electric focusing (IEF). The solution was composed of 7 M urea, 2 M thiourea, 0.5% Triton X-100, 4% CHAPS, 0.5% pharmalyte (pH 3-10), 0.1% NP-7 and 60 mM DTT. Protein concentration was determined (2-D Quant Kit, GE Healthcare, Uppsala, Sweden). Individual 11-cm immobilized pH gradient (IPG) strips, pH 3-10 NL (Bio-Rad, Hercules, CA) were rehydrated with samples followed by protein focusing (Protean IEF Cell, Bio-Rad). Focused proteins were reduced and alkylated and SDS-PAGE was performed on 12.5% precast polyacrylamide gels (Bio-Rad). Proteins were stained with Pageblue (Fermentas, Vilnius, Lithuania) overnight, quantified (GS-800-calibrated densitometer, Bio-Rad) and analyzed including determination of molecular weight and pI for the individual proteins (PDQuest Advanced 8.0.1, Bio-Rad). Proteins were identified by excision of spots followed by in-gel digestion with trypsin. Peptide masses were determined by mass spectrometry (MALDI-TOF MS) at the Wallenberg Consortium North Expression Proteomics Facility (Department of Medical Biochemistry and Microbiology, Uppsala University, Sweden) and proteins identified based on peptide masses.

### Western blot analysis

Samples for western blotting were prepared from MIN6 cells by washing the cells twice with PBS followed by lysing the cells on ice with a buffer composed of 150 mM NaCl, 20 mM Tris, 1% Triton X100, 0.25% Na-deoxycholate, 0.1% SDS, 1 mM Na_3_VO_4_, 2 mM EDTA and 1% protease inhibitor cocktail (Sigma P-8340) for 30 min. After lysis the preparations were collected and centrifuged at 13000 rpm for 15 min at 4°C. The supernatants were transferred to new tubes and the total protein concentration was determined by the DC protein assay (Bio-Rad) according to the manufacturer's instruction. Samples were mixed with SDS-PAGE sample buffer containing Tris-HCl (pH 6.8), SDS, glycerol and DTT and boiled for 5 min. Samples (25 μg per well) were then subjected to SDS-PAGE. After electrophoresis, proteins were transferred onto PVDF membranes. Immunoblot analyses were performed with antibodies towards phosphorylated eIF2α (Cell Signaling, Beverly, MA), phosphorylated AMPK (Cell Signaling), cleaved caspase-3 (Cell Signaling) and CHOP (Santa Cruz Biotechnology, Santa Cruz, CA). Immuno-reactive bands were imaged with Fluor-S MultiImager MAX (Bio-Rad) and quantified with Quantity One software (Bio-Rad). After imaging the PVDF membranes were stained with Coomassie and later de-stained with 50% methanol. The blots were then scanned in a standard table-top scanner and quantified with Quantity One software. The expression level of each protein was normalized to the Coomassie-stained blot.

### Fatty acid oxidation measurements

Reaction mixture was prepared by adding 2 μCi ^3^H-palmitate (GE Healthcare) per ml culture media containing 0.5% fatty acid-free BSA. Unlabelled palmitate was added to make the final concentration 0.25 mM. MIN6 cells exposed to fatty acids or not were washed with PBS. Cells were then incubated for 2 hours with the reaction mixture after which the media was collected. Radioactive water was separated from the radioactive palmitate in the media by three subsequent Folch extractions [31]. During extraction, proteins were isolated from the cells and the concentration was measured. After the last extraction, 10 ml scintillation liquid was mixed with the water phase and the mixture was counted in a scintillation counter. The results were normalized to protein amount in the corresponding wells.

### Glucose-stimulated insulin secretion

Glucose-stimulated insulin secretion (GSIS) was determined in MIN6 cells with reduced or control SCD1 levels. The cells were first incubated for 60 min in standard culture medium but with 2 mM glucose. Subsequently the medium was changed to KRBH buffer consisting of (in mM): glucose 2, NaCl 130, KCl 4.8, MgSO_4 _1.2, KH_2_PO_4 _1.2, CaCl_2 _2.5, NaHCO_3 _5.0, and HEPES 10, titrated to pH 7.4 with NaOH and supplemented with 1 mg/ml of BSA (fraction V, Boehringer Mannheim GmbH). The cells were allowed to rest for 30 min before medium was changed to the same type of buffer but with either 2 or 20 mM glucose. The cells were then incubated for 30 min. After incubation an aliquot of buffer was taken for later determination of released insulin. Cells were then washed in PBS, lysed in MilliQ H_2_O, and frozen for later determination of DNA content. Released insulin was determined with an ELISA as previously described [32].

### Data analysis

Results are presented as means ± SEM. Statistical significance between two conditions was analyzed by the Student's paired *t *test and between several groups using one-way ANOVA with Tukey post-hoc test. *P *< 0.05 was considered statistically significant.

## Competing interests

The authors declare that they have no competing interests.

## Authors' contributions

KT participated in the design of the study, carried out all the studies except for the 2D-gel part, analyzed the data and drafted the manuscript. MH performed the 2D-gel experiments and helped with the analysis of the data. PB participated in the study design and helped to draft the manuscript. All authors have read and approved the final manuscript.
